# Caval vein obstruction resulting in right to left shunt and desaturation post–endocardial pacing implantation in a 3-year old: a case report

**DOI:** 10.1093/ehjcr/ytae693

**Published:** 2024-12-24

**Authors:** Emma Poffley, Kristian Mortensen, Luke Starling, Jasveer Mangat, Jan Marek

**Affiliations:** Echocardiography Department, Great Ormond Street Hospital for Children, Great Ormond Street, London WC1N 3JH, UK; Echocardiography Department, Great Ormond Street Hospital for Children, Great Ormond Street, London WC1N 3JH, UK; Institute of Cardiovascular Sciences, University College London, London WC1E 6DD, UK; Echocardiography Department, Great Ormond Street Hospital for Children, Great Ormond Street, London WC1N 3JH, UK; Echocardiography Department, Great Ormond Street Hospital for Children, Great Ormond Street, London WC1N 3JH, UK; Echocardiography Department, Great Ormond Street Hospital for Children, Great Ormond Street, London WC1N 3JH, UK; Institute of Cardiovascular Sciences, University College London, London WC1E 6DD, UK

**Keywords:** Case report, Multi-modality imaging, Levo-atrial cardinal vein, Superior caval vein obstruction, Partial anomalous pulmonary venous drainage, Endocardial pacing

## Abstract

**Background:**

Superior caval vein obstruction is a rare complication of endocardial pacing lead implantation that can result in a right to left shunt.

**Case summary:**

A 3-year-old child with type 2 Brugada syndrome presented with mild cyanosis post–endocardial pacing implantation due to evolutionary right superior caval vein obstruction. This obstruction resulted in a right to left shunt across a previously unrecognized patent levo-atrial cardinal vein associated with partial anomalous pulmonary venous drainage. The patient underwent endocardial pacing explantation, balloon dilation and stenting of the right superior caval vein, banding of the levo-atrial cardinal vein to left upper pulmonary vein venous channel, and implantation of an epicardial pacing system.

**Discussion:**

Levo-atrial cardinal vein and partial anomalous pulmonary venous drainage with dual drainage can go undetected on cardiac imaging and may not ever cause symptoms (high left-to-right shunt or cyanosis). The levo-atrial cardinal vein and associated partial anomalous venous drainage with dual drainage was missed on multiple occasions and with multiple imaging modalities in our patient. Blood flow may not be detected in a small calibre or collapsed levo-atrial cardinal vein with pulmonary venous connection when the pulmonary vein remains widely patent and connected to the left atrium. Detailed comprehensive echocardiography of any child referred for cardiac intervention should include pulsed wave Doppler and colour flow mapping interrogation of the innominate vein from the suprasternal approach.

**Learning points**

- Children with isolated persistent levo-atrio cardinal vein and partial anomalous pulmonary venous drainage of the left upper pulmonary vein with dual drainage may never present with a significant left to right shunt and, therefore, may remain undetected during routine cardiac imaging, particularly echocardiography.
- Detailed comprehensive echocardiography of any child referred for cardiac intervention should include pulsed wave Doppler and colour flow mapping interrogation of the innominate vein from the suprasternal approach.

## Introduction

Benign cases of superior caval vein (SVC) obstruction are becoming more prominent^1^ with causes including venous catheters and pacing wires.^2^ Venous obstruction in children post–single lead endocardial pacing implantation has been reported at 13%–18%.^3,4^ Although often asymptomatic, SVC obstruction can present with head and arm swelling if insufficient collateral circulation develops.^5^

## Summary figure

**Table:** 

Date	Event
3rd August 2015	A 3-year-old child with type 2 Brugada syndrome presenting with profound reflex-anoxic seizures, loss of consciousness requiring CPR
5th August 2015	Endocardial single-lead pacemaker implantation via a brachiocephalic (innominate) vein
21st November 2018	Presented with symptoms of lethargy, breath-holding events, and appearing blue, the oxygen saturations were reported to be 89%–91%.
19th June 2019	A repeat sleep study showed no evidence of obstruction with ongoing low baseline saturations
2nd October 2019	Detailed echocardiogram including contrast study demonstrated a right-to-left shunt from the brachiocephalic vein to the left atrium
14th January 2020	A dedicated cardiovascular computed tomography angiogram confirmed a venous channel connecting the brachiocephalic vein, left upper pulmonary vein, and left atrium with a right-to-left shunt in the context of right superior caval vein obstruction
20th January 2020	The transvenous pacing system was explanted and replaced with a dual-chamber epicardial pacing system. The innominate vein to the left upper pulmonary vein venous channel was surgically banded
14th July 2021	Transcatheter balloon dilation of the residual stenosis at the junction of the right superior caval vein to the right atrium

Congenital vascular communications between the SVC territory and left atrium (LA) include persistent left superior caval vein (LSVC) with unroofed coronary sinus and levo-atrial cardinal vein (LACV). Levo-atrial cardinal vein is often associated with obstructive left heart disease, causing left-to-right shunting due to elevated LA pressure.^6^ Isolated left upper pulmonary vein (LUPV) partial anomalous pulmonary venous drainage (PAPVD) is often undiagnosed as the volume of shunting blood is too low to cause clinical symptoms and can be identified as an incidental finding on cardiac imaging. Cases of dual drainage of the LUPV with connection to both the LA and innominate vein via a LACV have been reported in structurally normal hearts, but are rare.^7–9^

We report the case of a child presenting with mild cyanosis post–endocardial pacing implantation due to evolutionary right SVC obstruction and right-to-left shunting across a previously unrecognized patent LACV.

## Case presentation

**Figure 1:**
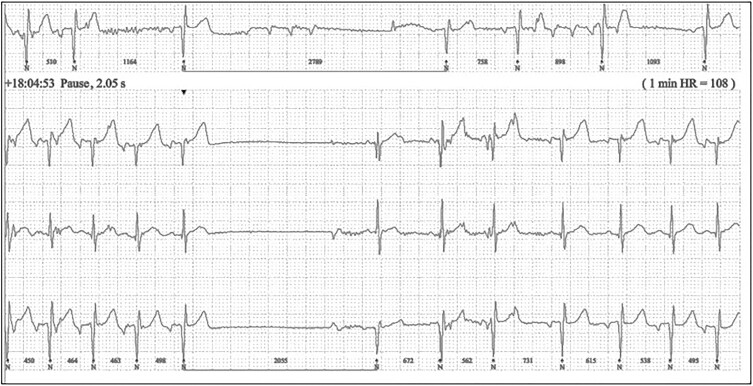
Initial 24-hour ECG revealed a number of pauses, the longest of which lasted in the region of 4.

A now 9-year-old female first presented at the age of 1 year with profound reflex-anoxic seizures, loss of consciousness requiring CPR on two occasions, and multiple episodes of apnoea. The patient has a family history of paternal sudden cardiac death during ajmaline provocation. The patient’s first episode occurred in the community with cardiorespiratory arrest requiring CPR. The second episode occurred overnight on the cardiac ward where prompt CPR took place and the patient fully recovered. The ECG of this second event demonstrated asystole with ventricular escape beats. The 24-hour tape showed Brugada type 2 and sinus pauses up to 4  (*Figure 1*). Subsequent genetic testing confirmed *SCN5A* mutation p.(Leu1222Arg).

A comprehensive echocardiogram at 17 months of age reported a structurally and functionally normal heart prior to her endocardial single lead pacemaker implantation. A left arm venogram showed a patent axillary vein and right SVC to right atrium (RA). An endocardial VVI pacing system was implanted via the left axillary vein with active fix lead (Capsure Novus 58 cm) to the RV apex and device (St Jude Microny) placed in the left axillary pocket. Echocardiogram post-implant showed no pericardial effusion, a stable lead within the RV and mild-to-moderate tricuspid regurgitation. The patient did not experience any further episodes of loss of consciousness following pacemaker implantation and was discharged home.

**Figure 2:**
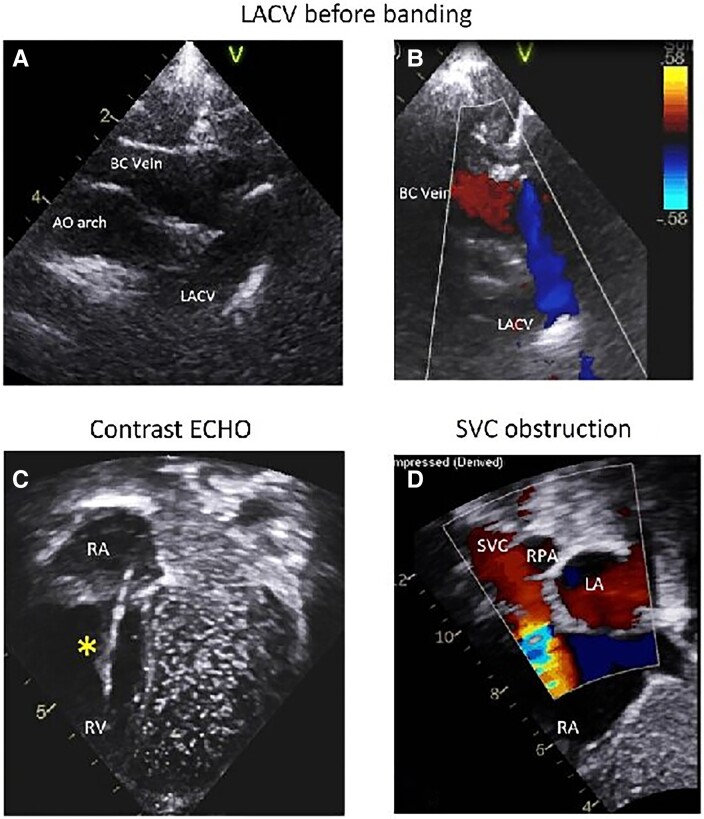
Composite echocardiographic images. Two-dimensional echo picture of brachiocephalic vein and connecting levo-atrial cardinal vein imaged from suprasternal view (*A*). Colour flow mapping indicates downstream flow from the levo-atrial cardinal vein towards the left atrium (*B*). Contrast echocardiography via left arm (*C*) demonstrated the right-to-left shunt from brachiocephalic vein into the left atrium. Endocardial ventricular pacing lead was seen within the right atrium and right ventricle (asterisk). Later, follow-up two-dimensional echocardiogram with colour follow mapping (*D*) performed after endocardial pacing system removal revealed obstruction of the superior caval vein at the level of cavo-atrial junction. LACV, levo-atrial cardinal vein; AO arch, aortic arch; LA, left atrium; RPA, right pulmonary artery.

**Figure 3:**
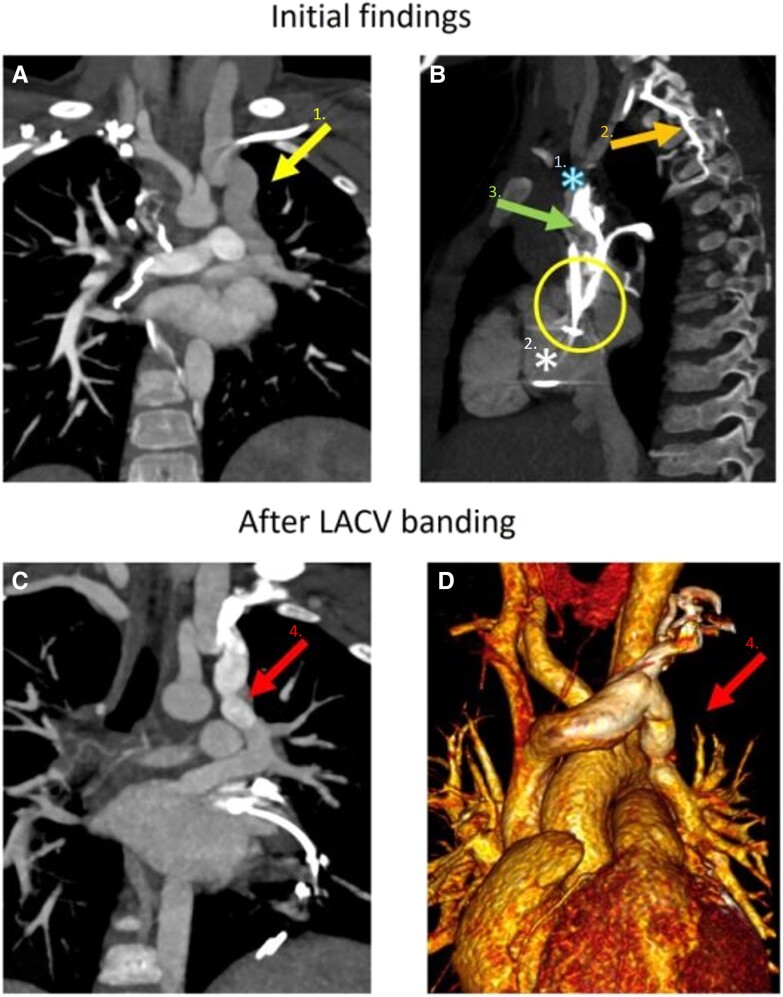
Composite computed tomography angiogram. The dedicated cardiovascular computed tomography (*A*) confirmed the presence of the levo-atrio cardinal vein (arrow 1), connecting the bridging vein to the left atrium via the left upper pulmonary veins. The computed tomography (*B*) also demonstrated hold up of contrast medium at the level of the superior caval vein to right atrium junction stenosis (circled), with contrast held up and delayed in the passage from the dilated superior caval vein (asterisk 1) into the right atrium (asterisk 2) and seeking into paravertebral collaterals and down the azygos vein (arrow 2). The pacing wire is seen *in situ*, running through the superior caval vein into the right atrium (arrow 3). A subsequent cardiovascular computed tomography (*C*, *D*) showed the banded levo-atrio cardinal vein (arrow 4). LACV, levo-atrial cardinal vein.

Three years later, the patient presented with symptoms of lethargy, breath-holding events, and appearing blue, oxygen saturations were reported to be 89%–91%. A sleep study showed no evidence of obstruction with ongoing low baseline saturations and a routine thoracic CT excluded lung disease and pulmonary embolus. The patient was referred for a detailed echocardiogram including contrast, this demonstrated a right-to-left shunt from the innominate vein to the LA (*Figure 2*). The right SVC was found to be obstructed by the pacing lead. A subsequent cardiovascular CT angiogram confirmed right SVC obstruction with an innominate vein to the LUPV venous channel and right to left shunt (*Figure 3*).

**Figure 4:**
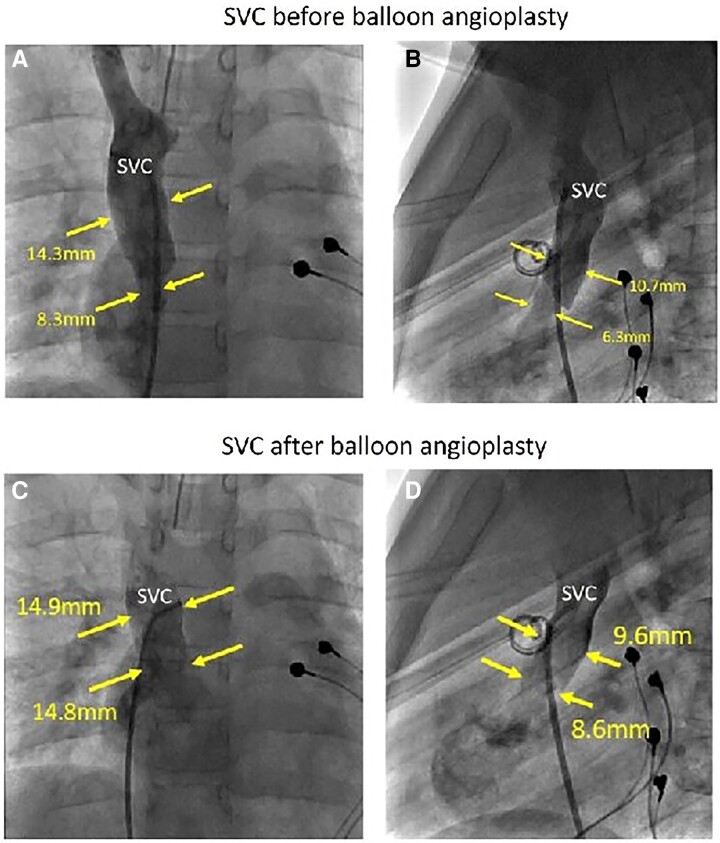
Composite X-ray cardiac angiogram in lateral (*A*) and antero-posterior (*B*) projection confirmed significant narrowing of the right superior caval vein at the level of the cavo-atrial junction. Angiogram after balloon angioplasty in lateral (*C*) and antero-posterior (*D*) projection confirmed successful dilatation with marked calibre change as compared with pre-procedural angiogram.

The patient underwent transvenous pacing lead explant and replacement with an epicardial pacing system. The innominate vein to LUPV venous channel was surgically banded. Six-month follow-up of the patient found residual obstruction to SVC flow with elevated central venous pressure and the patient underwent transcatheter balloon dilation of the stenosis at the junction of the right SVC and RA (*Figure 4*). The patient now remains clinically well with unobstructed flow in the right SVC to RA. There is a small persistent insignificant right-to-left shunt through the banded innominate vein to LUPV venous channel.

## Discussion

In this case report, we demonstrated a rare congenital abnormality unmasked by a complication following pacemaker insertion.

Multiple single centre reports have shown higher efficacy and safety of endocardial pacing than epicardial pacing systems in the paediatric population^10,11^ with fewer lead failure events and greater longevity in endocardial systems.^11^ Endocardial pacing systems are the favoured option in the paediatric population when the patient is not undergoing open surgery due to these favourable safety and longevity outcomes and less invasive procedure.

Superior caval vein obstruction due to pacing wire and subsequent right-to-left shunting are extremely rare complications. A retrospective analysis of the thoracic CT performed prior to pacing implant was reviewed in detail. This scan showed a pattern of contrast enhancement suggestive of communication between the SVC, innominate vein, and LUPV missed on multiple imaging modalities. The morphology was similar to that demonstrated in two case studies. The first documents a 72-year-old male with post-surgical complete heart block requiring implantation of a transvenous pacemaker.^12^ He presented with shortness of breath, cyanosis, and reduced oxygen saturations, bubble echo demonstrated opacification of the LA and subsequent cardiac catheterization and MRI demonstrated SVC stenosis and decompression of the upper body via an anomalous venous channel to the LA.^12^ The second case documents a 69-year-old patient with previous bicuspid aortic valve replacement, cardiovascular CT demonstrated a previously undiagnosed partial anomalous connection of the LUPV with dual drainage to the vertical vein and LA.^13^

The right-to-left shunt described in our case, innominate vein to LUPV venous channel, requires differentiation between LSVC, LACV, and PAPVD of the LUPV. Both LSVC and LACV present as venous channels running lateral to the aortic arch, however, have different embryological origins.^14^ Although a LSVC is much more common, 0.3%–0.5% of the general population,^14^ it can almost certainly be ruled out in the presence of drainage to the LUPV and an intact coronary sinus.^14^ Levo-atrial cardinal vein is predominantly noted in left-sided obstructive disease, however, has rarely been identified in structurally normal hearts. The embryological origin of the LACV is via the capillary plexus surrounding the embryological foregut, this plexus develops into the pulmonary veins connecting to the left atrium. During this developmental period, the capillary plexus is connected to both the future pulmonary veins and the cardinal venous system, this connection is gradually lost. Levo-atrial cardinal vein is the persistence of this connection to the cardinal venous system.

**Figure 5:**
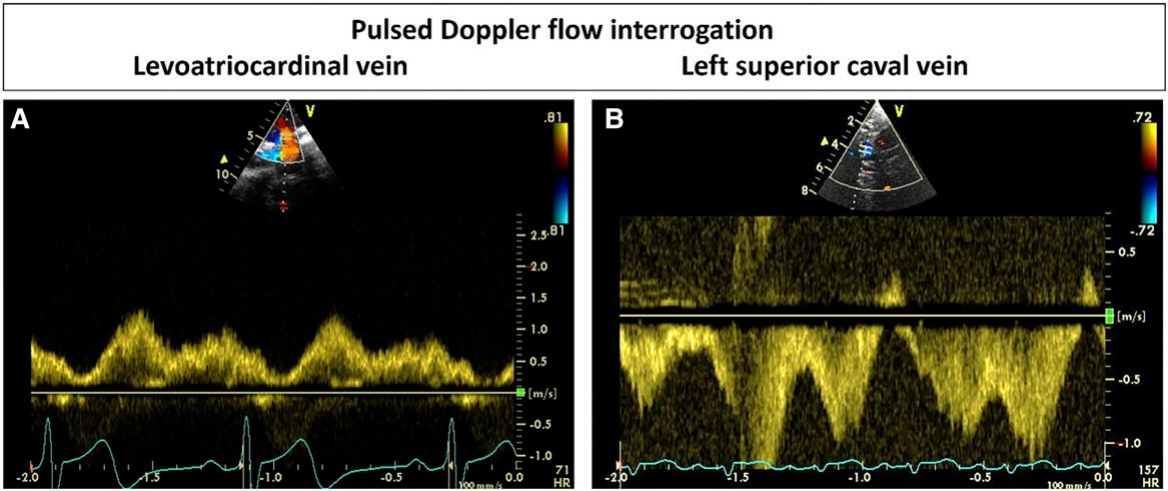
Illustrative examples of pulsed Doppler interrogation in (*A*) a patient with levoatrial cardianal vein in association with obstructive left heart disease and (*B*) a patient with persistent left superior caval vein draining coronary sinus.

In our case, the SVC obstruction was not immediately detected by routine transthoracic echocardiography. Later contrast echocardiography identified the right-to left shunt prompting detailed interrogation of the SVC. Computed tomography angiogram or CMR are non-invasive gold standard procedures for assessment of SVC obstruction with venography being the invasive gold standard.^15^ However, echocardiography utilizing pulsed-wave Doppler (PWD) and colour flow mapping (CFM) is accurate in the detection of SVC stenosis in patients with pacemakers,^15^ and all echocardiograms for patients with endocardial pacing leads should include PWD and CFM interrogation of the SVC (*Figure 5*). Two-dimensional echocardiography combined with CFM and PWD interrogation of any abnormally connected vessels to the innominate vein should help to differentiate between systemic (LSVC, hemiazygos vein, venous collaterals, LACV) and pulmonary (PAPVD) venous connections.

Children with isolated LACV and PAPVD of the LUPV with dual drainage may never present with a significant left to right shunt and, therefore, may remain undetected during routine cardiac imaging. We believe that echocardiographic imaging of the innominate vein, via suprasternal approach, should be a routine prior to any cardiac intervention and should be included in all institutional, national, and international paediatric guidelines.

## Lead author biography


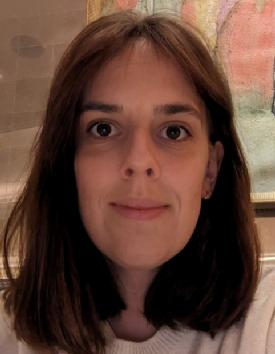


Emma Poffley is a cardiac clinical scientist at Great Ormond Street Hospital. Emma completed the scientific training programme at Oxford University Hospitals and Manchester Metropolitan University, before working in the echocardiography department at Royal Papworth Hospital. Emma then moved to Great Ormond Street Hospital to specialize in paediatric congenital echocardiography.

## Supplementary Material

ytae693_Supplementary_Data

## Data Availability

The data underlying this article are available in the article and in its online Supplementary material.

## Supplementary material

**Consent:** The authors confirm that written informed consent for submission and publication of this case report including images and associated text has been obtained from the patient’s next of kin in line with COPE guidance.

**Funding:** None declared.
